# Physiological and pathological effects of phase separation in the central nervous system

**DOI:** 10.1007/s00109-024-02435-7

**Published:** 2024-03-05

**Authors:** Jiaxin Wang, Hongrui Zhu, Ruijia Tian, Qian Zhang, Haoliang Zhang, Jin Hu, Sheng Wang

**Affiliations:** 1 Department of Anesthesiology, First Affiliated Hospital of USTC, Division of Life Sciences and Medicine, University of Science and Technology of China, Hefei, Anhui 230001 People’s Republic of China; 2 School of Medicine, Xiamen University, Xiamen, Fujian 361000 People’s Republic of China; 3 Core Facility Center, The First Affiliated Hospital of USTC (Anhui Provincial Hospital), Hefei, China

**Keywords:** Phase separation, Physiological function, Pathological effect, Neurodegenerative disease

## Abstract

Phase separation, also known as biomolecule condensate, participates in physiological processes such as transcriptional regulation, signal transduction, gene expression, and DNA damage repair by creating a membrane-free compartment. Phase separation is primarily caused by the interaction of multivalent non-covalent bonds between proteins and/or nucleic acids. The strength of molecular multivalent interaction can be modified by component concentration, the potential of hydrogen, posttranslational modification, and other factors. Notably, phase separation occurs frequently in the cytoplasm of mitochondria, the nucleus, and synapses. Phase separation in vivo is dynamic or stable in the normal physiological state, while abnormal phase separation will lead to the formation of biomolecule condensates, speeding up the disease progression. To provide candidate suggestions for the clinical treatment of nervous system diseases, this review, based on existing studies, carefully and systematically represents the physiological roles of phase separation in the central nervous system and its pathological mechanism in neurodegenerative diseases.

## Introduction

The interior and exterior of the classical organelle are physically distinct because the cell membranes typically prevent biomolecules from passing through, and the composition of the organelle is controlled by the unique membrane transport system [1]. However, some cell compartments are not bound to the membrane. Montgomery and Wilson discovered membrane-free chambers in the 1930s [2]. As the name suggests, there are a number of membrane-free compartments within cells that either lack membranes entirely or are not surrounded by the membrane [3]. Additionally, many of them are produced by proteins binding to nucleic acids or other polypeptides that function as polymeric scaffolds [4]. That dynamic aggregation of biological macromolecules in cells is regarded as phase separation. According to multiple studies, phase separation could be a mechanism that directs the creation of membrane-less organelles or recruits proteins into membrane-less organelles [5]. For example, phase separation offers cells an isolated chamber that can not only accelerate the reaction but also prevent the interference of irrelevant substances in the cytoplasm or nucleus [6]. The normal occurrence of enzymatic reactions in cells depends critically on phase separation. As such, enzymes and their substrates are assembled into a specific location at a certain time to finish a series of biological processes [6]. Furthermore, subcellular contents are spatiotemporally organized in response to environmental cues for physiological functionality [7].

There is growing evidence demonstrating the dynamic formation process of various membrane-less organelles in living cells, which is significantly influenced by phase separation, particularly liquid-liquid phase separation (LLPS) [8]. LLPS is a reversible process by which a given molecule spontaneously separates into two phases, a dilute phase and a highly concentrated and condensed phase, when a molecule disperses uniformly in the liquid state after reaching a necessary threshold concentration [9, 10]. Biomolecules dwell in phase-separated droplets, like oil drops in water, in their concentrated and diluted states [11]. The molecular underpinning of LLPS is the emergence of multivalent and low-affinity interactions, including electrostatic, hydrophobic, and other affinities [12]. Notably, the impacting factors include critical concentration as well as intracellular external conditions like temperature, pH, and ionic strength [13–16]. Moreover, it has been proposed that all biopolymers can undergo LLPS when subjected to certain conditions [1, 17].

The physical LLPS mechanism aids in the formation of many membrane-free organelles. These membrane-less organelles are called biomolecular condensates. Condensates, such as stress granules (SGs), processing bodies (P-bodies), Cajal bodies, and nucleoli, have recently provided increasing proof of playing critical roles in biological activities [2]. Since they lack membranes, these feasible roles undergo activation and deactivation in response to minute environmental changes, such as temperature or pH variations, via the emergence and disassembly of the condensates [18, 19]. Additionally, their membrane-free quality is closely related to the quick movement of molecules across phases, allowing for unhindered transit through the barrier and a rapid chemical equilibrium between compartments with various properties. For example, a stable protein concentration gradient in the cytoplasm can be produced by spatially separated phosphorylation and dephosphorylation processes, which can then influence the protein diffusion rate [20]. While modulation to the protein concentration gradient may allow for dynamic assembly and disassembly of membrane-free organelles [21, 22], the interaction of molecules inside droplets and the surrounding fluid is visible through fluorescence recovery after photobleaching (FRAP). The rapid breakdown of liquid compartments may result from the entry of proteins that are concentrated in protein droplets or other regulators into droplet phases [23]. Likewise, enhanced thermodynamic and kinetic favorability of specific reactions could occur in some droplets as a result of a little rise in component concentration [24]. Notably, reactions in the cytoplasm may halt as cytoplasmic constituents become exhausted when they segregate into the condensed phase [25]. Biomolecular condensates are viewed as a quicker spatially structured and compartmentalized regulated form of intracellular substance as a result of their rapid responsiveness [26]. Many of these structures are common to a large proportion of cells, like the membrane-less structures located within the nucleus. The nuclear bodies nucleolus, Cajal bodies, and promyelocytic leukemia nuclear bodies (PML NBs) are just a few of the many nuclear bodies that have been defined thus far [13]. Cajal bodies have been thought to be involved in the ribonucleoprotein assembly process, which is necessary for housekeeping functions within the nucleus [27]. In addition to contributing to the subcellular localization and functional regulation of certain ribonucleoprotein telomerase components, Cajal bodies are also involved in cellular stress responses like RNA interference and DNA damage repair pathways [27]. PML NBs, a macromolecular polyprotein complex, have the property of phase separation of liquid droplets. PML NBs could interact with chromatin and alter the availability of chromatin-related components by regulating transcription factor activity [28]. Nevertheless, the LLPS-driven membrane-less compartment exists as well in the cytoplasm. For example, the P-bodies and SGs are composed of mRNAs and proteins and include translation initiation-stalled mRNA-protein complexes (mRNPs). Moreover, eukaryotic cells produce stress granules, which are condensates as a response to stress [29, 30]. Numerous non-translated mRNAs, translation initiation factors, and several proteins that influence mRNA functions are found in it. Researchers discovered that protein and RNA spontaneously condense on one side of the cell to produce P granules. Importantly, this droplet-like structure can exhibit fusion, wetting, flow, and other liquid characteristics. P granules rapidly dissolve or consolidate as the concentration of linked components changes [31]. As an illustration, consider the sequestration of mTORC1, which is stored in structures that resemble P granules and are known as stress granules. Consequently, stress granules disintegrate as a result of DYRK3 kinase activity, releasing mTORC1 for signaling [22].

The existence of phase separation in cells allows compartments with diverse chemical characteristics to maintain chemical balance via the fast movement of molecules, participating in RNA metabolism, ribosome biosynthesis, DNA damage response, and signal transduction [32]. Currently, there are three possibilities in which the abnormalities of phase separation can cause illness. Genetic mutations or environmental effects may impair the production of condensate by (a) directly modifying the molecular process of condensate assembly, (b) influencing the activity of a master regulator of condensation, or (c) changing the fundamental physicochemical circumstances within cells [33]. Many neurodegenerative diseases, including amyotrophic lateral sclerosis (ALS), Alzheimer’s disease (AD), Huntington’s disease (HD), and Parkinson’s disease (PD), are connected to LLPS disorders. Consequently, this paper covers the physiological functions of phase separation, their significance for the central nervous system, and the pathological underpinnings of illnesses in the central nervous system. Notably, phase separation could provide researchers with a novel outlook for comprehending and treating human diseases.

## The physiological functions of phase separation

Phase separation can provide independent units for the occurrence of enzymatic reactions, protect the orderly progression of enzymatic reactions, and facilitate intracellular transcription regulation [34]. LLPS plays indispensable roles in biological processes, like signaling transduction, gene regulation and transport, DNA damage repairing, and inter-synaptic signaling [2].

### Transcriptional regulation

In eukaryotes, transcriptional regulation is an important mechanism in regulating gene expressions. Enhancers are cis-regulatory elements of the genome that influence expression patterns of target genes in a tissue- and cell-type-specific manner [35]. “Super-enhancers” refer to an expansive domain constituted of collections of enhancer elements linked to critical cell-type-specific genes and cell identities, which are essential to the biological functions of these cells [36, 37]. In vitro studies have revealed that cyclin T1 (CycT1) and dual-specificity tyrosine phosphorylation-regulated kinase 1A (DYRK1A) could combine to create droplets [38]. In contrast, the histidine-rich domain (HRD) could recruit polymerase (Pol) II into the droplets by interacting with the C-terminal domain (CTD) of the largest subunit of RNA polymerase II of human RNA Pol II, promoting phosphorylation and transcriptional elongation of Pol II [38]. Octamer-binding transcription factor 4 (OCT4) is a crucial transcription factor in embryonic stem cells (ESCs), containing a DNA binding domain and a transcriptional activation binding domain. OCT4 interacts with the transcriptional mediator complex subunit 1 (MED1) through the transcriptional structural domain to form phase-separated condensates at super-enhancers of ESCs, stimulating the gene expressions [39]. Phase separation, related to genomic activity, interacts with autosomal regions with lower chromatin density. Sanulli demonstrated that Swi6, pombe HP1 protein, significantly boosts the accessibility and dynamics of hidden histone residues within a nucleosome, which contribute to dynamic exposure of buried nucleosomal regions and reducing these dynamics could hamper the ability of Swi6 to condense chromatin into liquid droplets [40]. Eukaryotic chromatin is heavily condensed and divided into chromosomes and flexibly accessible to being regulated. Similar to how chromatin acts in cells, eukaryote-wide histone H1 and inter-nucleosome linker lengths drive chromatin phase separation, regulate droplet properties, and interact together to generate condensates of uniform density [41]. Additionally, chromatin phase separation was capable of being de-antagonized by histone acetylation of protein p300, resulting in droplet lysis in vitro and fewer droplets in the nucleus [41]. These findings indicated that phase separation was vital in the regulation of the transcription process in organisms. Furthermore, phase separation condensates in mitochondria can modulate DNA transcription [42]. Phase-separated condensates of mitochondrial transcription factor A (TFAM) will aggregate to generate circular mitochondrial DNA (mtDNA), resulting in TFAM-mtDNA droplets that significantly concentrate transcriptional enzymes for effective transcription, including TFB2M and mitochondrial RNA polymerase (POLRMT), moreover, a co-phase separation that can suppress transcription is constituted of TFAM-mtDNA and mitochondrial transcription termination factor (MTERF1) [42].

### Signal transduction

Phase separation occurs frequently in the downstream signal transduction pathways and the molecular mechanism of cell-surface receptors. Transmembrane receptors on the surface of cells typically cluster from the nanometer to the micrometer scale to initiate signal transduction [43, 44]. Phase separation is a critical cellular mechanism of activity on biological membranes [45]. Transitions between ordered and disordered phases are feasible in lipid bilayer model membranes, and phase separations can occur in membranes comprised of multiple lipid species [46]. Multiple complex components within the cell membrane form a certain order in a phase-separated form to regulate the physiological functions of the cell so that the corresponding cellular functions can be performed at the appropriate time and space. Lipid phase separation in membranes impacts the transport of lipids and proteins as well as the local composition, dynamics, and allosteric modulation of membrane proteins [47]. Moreover, protein–protein collisions cause steric pressure [48], destabilizing the lipid phase separation and causing proteins and lipids to be evenly distributed on the membrane surface, maintaining the dynamic balance of the components of membrane.

LLPS has been proven to facilitate the clustering of transmembrane proteins with their cytoplasmic binding partners at membranes [49]. Linker for the activation of T cells (LAT) includes several tyrosines (Y) that are phosphorylated following T cell receptor (TCR) triggering. Three of them (Y171, Y191, and Y226) are known to attract the cytosolic adaptor protein, growth factor receptor-bound protein 2 (Grb2), via their Src homology 2 (SH2) domains. Moreover, Grb2 has two SH3 domains that interact with proline-rich areas in the nucleotide exchange factor’s C-terminal domain, Son of Sevenless (SOS) [49]. They can interact with the phosphorylated LAT and engage in phase separation [50], which promotes downstream signaling pathways. Notably, phase separation occurs in the nephrin/non-catalytic region of tyrosine kinase (Nck)/neural Wiskott-Aldrich syndrome protein (N-WASP) [51] system during membrane signaling in renal podocytes, and the degree of phase separation is regulated by the relative concentrations between molecules. Phase separation also significantly up-regulates the activity of N-WASP by increasing its membrane dwell time and enhances its ability to stimulate actin-related protein 2/3-mediated actin assembly. SOS is a guanine nucleotide exchange factor (GEF) in the LAT-Grb2-SOS signaling pathway, an essential Ras activator that is autoinhibited in the cytosol [52]. Huang et al. identified several phases involved in the process of full-length SOS stimulating the Ras GEF activity. After Grb2 recruits SOS to the membrane, the dwell time is approximately 4 ~ 6 s on the membrane. Phase separation can be observed to merge with rising SOS concentrations and then repel SOS to make it leave the cell membrane rapidly [53]. Following Grb2-mediated membrane recruitment, structural changes within SOS uncover the allosteric Ras-binding site and free autoinhibition of SOS guanine nucleotide exchange activity. In this process, the SOS condensate produced by phase separation could stay on the membrane for a considerable time [50]. This ensures that the interference signal in the upstream signal pathway will not significantly activate the downstream signal pathway under the conditions of low activity level. G protein-coupled receptor kinase interactor (GIT) and PIX are Arf-specific GTPase-activating proteins (GAPs) and Rho-specific guanine nucleotide exchange factors (GEFs), respectively. The GAP-ANK-SHD sequence of the GIT and PIX was able to form high-concentration condensate GIT2/β-Pix, which improved the ability to activate the GTP enzyme [54]. Furthermore, the multivalent interaction developed in the cytoplasm allowed for this phase separation behavior. GIT/PIX condensates can also target various cellular compartments via phase separation [54].

Many studies have illustrated how the interaction between the GIT1/β-Pix complex with paxillin promotes phase separation [55]. Through GIT1-mediated binding to paxillin, GIT1/β-Pix condensates generated by endogenous enzymes can be directed to focal adhesions, playing a critical role in regulating cell migration. When Shank family scaffold proteins bind to β-Pix in neurons [56, 57], β-Pix is concentrated at postsynaptic densities (PSDs) of excitatory synapses to regulate cell growth and synapse development. By serving as a scaffold complex [58], PIX and GIT regulate the activity of the Hippo (Hpo) kinase and encourage Hpo dimerization and autophosphorylation of the Hpo activation loop. Additionally, Hippo is the critical regulator of the Hippo pathway [59]. Mammalian Ste20-like kinases 1/2 (MST1/2, homologues of Hpo) and mitogen-activated protein kinase kinase kinase kinase (MAP4K) family members contribute to phosphorylate large tumor suppressor 1/2 (LATS1/2, homologues of Wts)/transcriptional co-activator with PDZ-binding motif (TAZ)[60]. Yes-associated protein (YAP)/TAZ is phosphorylated and inhibited by LATS1/2 to regulate cell growth and proliferation. The Laforin-Mst1/2 complex is assembled due to LLPS occurring during the aggregation of excessive glycogen, inducing excessive cell growth and proliferation as well as inhibiting apoptosis [61]. Phase separation is also necessary for insulin signaling to initiate the metabolism of glucose and glycogen. Insulin receptor substrates 1/2 (IRS) are known to recruit phosphatidylinositol 3-kinase (PI3K)-3-phosphoinositide-dependent protein kinase-1 (PDK1)-protein kinase B (PKB) to the plasma membrane after developing phase separation in the cytoplasm and nucleus [62]. Consequently, PKB can detach from the plasma membrane since being phosphorylated by PDK1 and the mechanistic target of rapamycin C2 (mTORC2) to access the cytosol and nucleus and phosphorylate its downstream targets. Accumulating results indicate that the phase separation of biomacromolecules can activate membrane receptors and their downstream signals [63]. Researchers have a great demand for effective and safe phase separation tools. The “biomimetic protein phase separation technology” designed and implemented by Li et al. [64] may flexibly manage and visualize the aggregation of cell membrane receptors and the subsequent signaling of those results. This is anticipated to lead to the development of a new paradigm for initiating receptor multimerization.

### DNA protection and damage repair

Defects in the DNA damage response (DDR) can induce genomic instability, potentially leading to tumors and neurodegeneration [40]. There are three major phases in the repair of DNA damage: (1) Firstly, DNA damage sites are sensed and recruited by intracellular DNA damage sensor proteins such as poly (ADP-ribose) polymerase 1 (PARP1) and MRE11-RAD50-NBS1 (MRN) complex. (2) Subsequently, these proteins further recruit downstream signal sensor proteins such as ataxia-telangiectasia mutated and rad3 related (ATR) and ataxia-telangiectasia mutated (ATM), meanwhile amplifying the signal. (3) Ultimately, activated effector proteins, such as p53, arrest the cell cycle and repair damaged DNA through DNA repair pathways including non-homologous end joining (NHEJ) and homologous recombination (HR) [65]. p53-binding protein 1 (53BP1) contains an intrinsically disordered region (IDR) at its C-terminus, which is the molecular structural basis of phase separation. Kilic et al.’s team confirmed that 53BP1 facilitates the assembly of downstream effectors and draws DNA damage repair proteins by optoDroplet experiments [66]. 53BP1 acts as master scaffold to generate functional complexes with double-strand break (DSB)–responsive factors at damaged chromatin. Through interactions with the MRN complex, DSBs trigger the stimulation of ATM and ATM-induced 53BP1 phosphorylation to phase separation [66, 67]. Non-POU domain-containing octamer-binding protein (NONO) can undergo spontaneous phase separation in vitro, which is recruited to DNA damage sites and participates in the NHEJ DNA damage repair process [68]. Furthermore, condensate formation through phase separation of NONO protein can recruit epidermal growth factor receptor (EGFR) and DNA-dependent protein kinase (DNA-PK) into condensate and enhance the interaction between EGFR and DNA-PK, promoting phosphorylation of T2609-DNA-PK and thus enhancing NHEJ-mediated DNA damage repair [69].

## Phase separation mediates synaptic-related physiological activities

### Formation of presynaptic active region complex

It was observed that in neurons, electron-dense particles appear in presynaptic zones visualized by electron microscopy. Optical super-resolution microscopy allowed imaging of Rab3-interacting molecule (RIM), RIM binding protein (RIM-BP), and ETS-like transcription factor (ELKS) to form dense clusters in the active region. Notably, the deletion of RIMs and RIM-BP together significantly reduces the ability of active zone–specific presynaptic dense projections to assemble [70]. Wu et al. further demonstrate that the main components of presynaptic active region complex are a set of scaffold proteins related to synaptic vesicles (SVs) and fusion, including, RIM, RIM-BP, ELKS, and calmodulin-dependent serine protein kinase (CASK). Importantly, they could form condensates through phase separation [71–75]. Likewise, the recombination method in vitro confirmed that the purified RIM and RIM-BP mixtures could be phase separated at physiological concentrations. Here, researchers showed how phase separation allowed RIM and RIM-BP to build dynamic and condensed assemblies through multivalent bindings. The specific mechanism was that RIM1a-PRMs (proline-rich motif; “S” for SH3 domain binding) interacts with all three Src homology 3 (SH3) domains of RIM-BP, through C-terminal-tail-mediated directly binding to both RIM and RIM-BP [76]; voltage-gated Ca^2+^ channels (VGCCs) could be enriched to the RIM and RIM-BP condensates [71]. Finally, VGCCs-RIM-BP-RIM facilitated phase separation. The scaffold protein condensate between ELKS and Liprins was similar to RIM/BP-RIM, which also anchored to the presynaptic membrane by binding to proteins [71].

### Formation of postsynaptic dense density

**Fig. 1:**
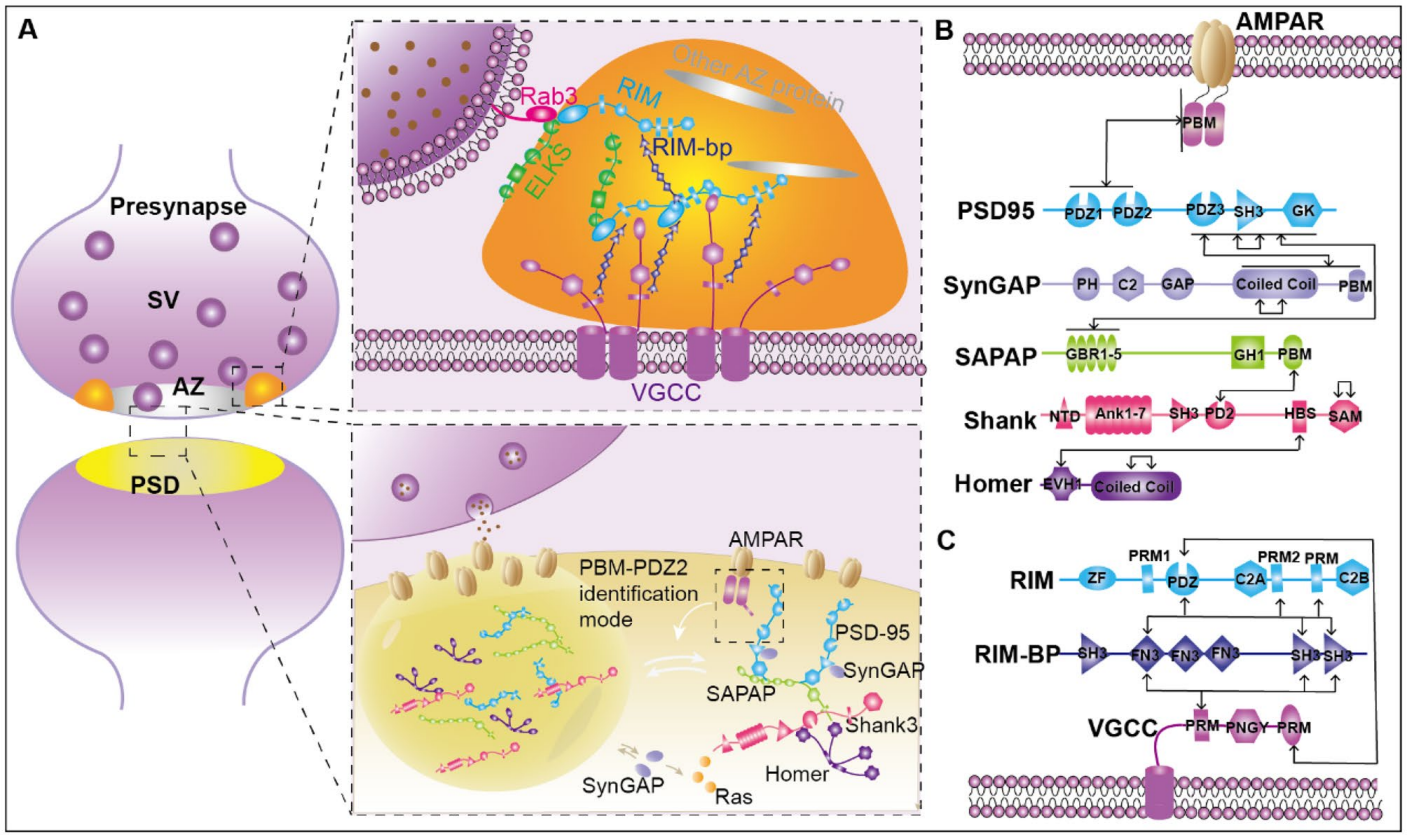
Phase separation at the synapse. **A** Formation of presynaptic active region complex and postsynaptic dense density. **B**, **C** Domain organization and interaction network of major scaffold proteins mediating phase separation of the ePSD (**B**) and the active zone (**C**). Two-way arrows denote interactions between proteins within each network and self-associations of domains. Other types of binding are indicated by one-way arrows. AZ active zone, PSD postsynaptic density, SV synaptic vesicle

Protein-rich subcompartment, the postsynaptic density (PSD), an aggregation responsible for receiving, decoding, and storing signals sent by presynaptic axonal termini, is located beneath the postsynaptic plasma membranes of each synaptic junction [77]. Synaptic strength was positively correlated with the number of proteins in the PSD of the postsynaptic membrane. The main components of excitatory PSDs (ePSDs) are scaffold proteins, including PSD-95, SAP90/PSD-95-associated protein (SAPAP), Shank3, and Homer [78–81]. ePSDs can enhance the stability of synaptic structure and provide structural support for functional enzyme SynGAP. Gephyrin-binding proteins in inhibitory PSDs (iPSDs) are neither highly accumulated in synapses like gephyrin nor are multidomain scaffold proteins [82]. Most iPSDs are thought to reside on the dendritic shaft, while ePSDs are mainly confined to the protrusions along the dendritic spines [83]. Additionally, the scaffold protein in PSD is highly concentrated and moves dynamically. However, its diffusion rate is much slower, indicating that the phase separations of the scaffold protein and PSD are usually correlated. Zeng and team’s in vitro experiments found that when scaffold proteins PSD-95, SAPAP1, Shank3, and Homer3 were mixed at a molar ratio of 1:1:1:1, the mixture would phase separate and these four proteins well co-localized and condensed into spherical droplets, resulting in a decreasing speed [84]. In vivo experiments of mice also demonstrated that the multivalent interaction between transmembrane AMPA receptor regulatory protein (TARP) and PSD-95 in TARP gamma-8 mutant decreases synaptic transmission function in hippocampal neurons, proving that ePSD formation mediated by phase separation can functionally promote AMPAR aggregation and synaptic transmission [76]. Epilepsy, intellectual disability (ID), and autism spectrum disorder (ASD) are all correlated with SynGAP mutations [85]. Furthermore, SynGAP acts non-enzymatically in PSD and synaptic activity is highly sensitive to the dose of SynGAP. When synapses are stimulated, SynGAP disperses from PSD condensate in living neurons. Thus, activation of small G proteins such as Ras is a negative modulator of synaptic strength. Therefore, in vitro experiments showed that the homotrimer of SynGAP and PSD-95 could undergo phase separation, but the monomer SynGAP could not generate LLPS, but not the monomer SynGAP [86] (Fig. 1).

## Phase separation participates in neuronal development

### Symmetric cell division

The mitotic spindle is an organelle rich in microtubules (MTs) and is essential for the accurate segregation of sister chromatids into daughter cells. The mitotic spindle performs functions depending on three key components: spindle poles, spindle microtubules, and kinetochore microtubules [87]. The poles of the mitotic spindle arise from centrosome amplification, while meiotic spindle is the absence of centrosome [88]. The centrosome serves as the primary microtubule-organizing center (MTOC) in most animal cells [89]. Centrosomal proteins regulate centrosome maturation and separation during phase separation. Polo-like kinase 4 (Plk4) assemblies concentrate tubulin and act as an MTOC to promote centrosome maturation and stability. During centrosome formation and maturation, Plk4 is firstly localized to centriole by exhibiting a ring-like pattern of localization around the Cep152 scaffold in early G1. Plk4 can also phase separate and construct a spherical condensate by using its autophosphorylated non-catalytic cryptic polo-box (CPB). Likewise, Plk4 condensate can behave as an assembly element for centriole biogenesis since CPB phosphorylation encourages Plk4 to escape from the Cep152 tether while binding to downstream STIL [90]. Notably, when the centrosome proteins Plk1, SPD-2 (mammalian homologue: Cep192), TPXL-1 (mammalian homologue: TPX2), and Zyg-9 (mammalian homologue: CKAP5) were incorporated into the centrosome surfactant-associated protein D-5 (SPD-5) condensate, the phase separation of the condensate was enhanced. As mammalian oocytes lack traditional centrosomes, the activity of transforming acidic coiled-coil protein-3 (TACC3) and its related receptors and enzymes is reduced [91]. Thus, these effects will destroy the liquid-like meiotic spindle domain (LISD), resulting in significant diminishing of K-fibers and interpolar microtubule density, as well as spindle volume, and the delay in separation [92].

### Asymmetric cell division

Neuroblast (NB) is the main member in adult neural stem cells, which proliferates into neural precursor cells, neurons, and various glial cells [93]. Once NBs transform into an asymmetric cell division cycle, they will yield an apical–basal structure. Importantly, NBs divide into a larger cell that retains NB characteristics and a smaller ganglion mother cell (GMC) through interaction between protein aggregates and stellate microtubules, which is crucial in nervous system development [94]. In the asymmetric cell division (ACD) of Drosophila neural stem cells, related complexes are formed to establish of cell apical–basal polarity. When proteins and RNA within each pole split apart obliquely, this enables daughter cells of different fates. Moreover, these complexes begin to assemble at the interphase of mitosis and agglutinate at the metaphase and dissolve at the anaphase. This dynamic process indicates that the development of these complexes is closely related to phase separation [95]. Previous in vivo and in vitro research on Numb and Pon showed that phase separation is the driver of Numb polarization condensation. Multivalent Numb/Pon interaction network in vitro shows that many spherical droplets can rapidly fuse into larger droplets, and the formation of condensate is characterized by phase separation [96]. During ACD process, Par protein complexes Par3, Par6, and aPKC are also assembled by phase separation and then regulate the establishment of top–bottom cell polarity axis and neuron differentiation [96].

## Pathological roles of phase separation in the central nervous system

Liquid-like droplets gradually transform into irreversible solid in pathological conditions during aging. Additionally, aged condensate droplets do not fuse but are more prone to clumping and sometimes forming a network of amyloid fibrils and amyloid-like aggregates [97]. Many neurodegenerative disorders are caused by aggregation and deposition of abnormal condensates [98]. As such, neurodegenerative diseases are characterized by progressive loss of cognitive and motor function, such like AD, PD, ALS, HD, frontotemporal lobar degeneration (FTLD), and traumatic brain injury [99]. Thus, identifying the cause and mechanism of protein-aggregation is crucial for precision therapy. Importantly, the concept of phase separation represents a new research idea for studying the disorder of protein self-assembly in neurodegenerative diseases.

### AD

**Fig. 2:**
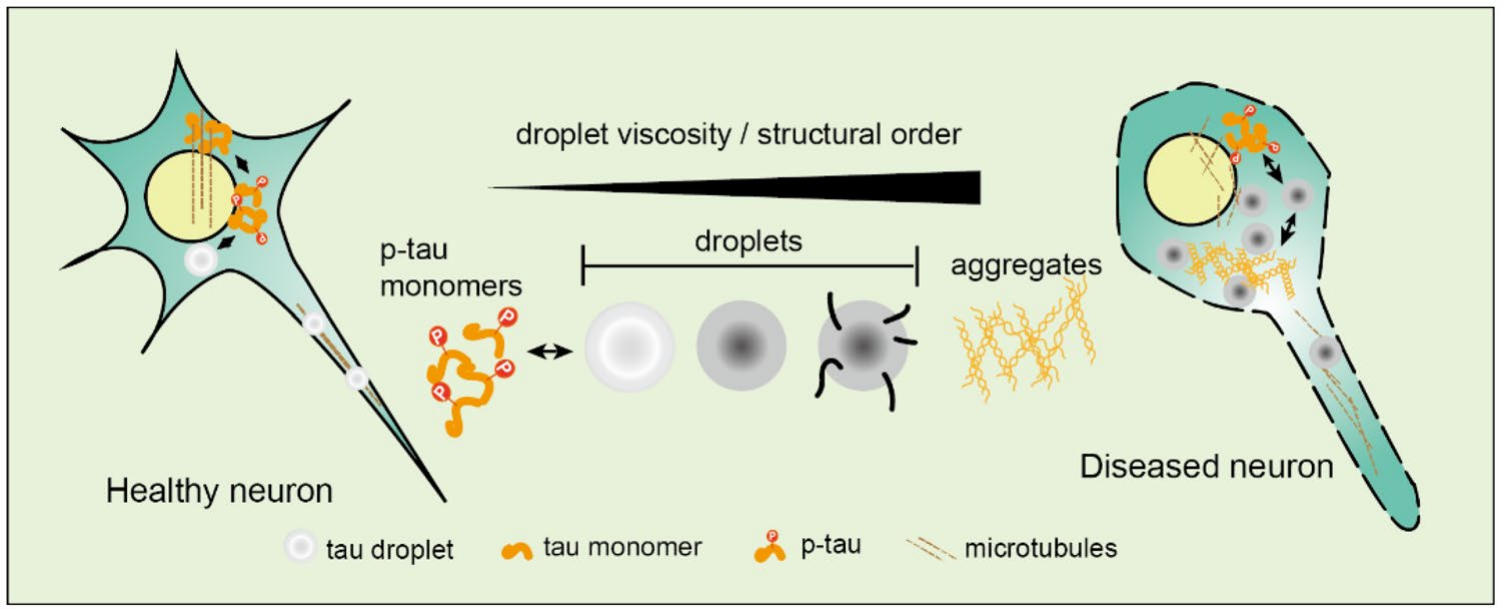
Phase separation of tau protein in neurons. In healthy neurons, physiological tau phosphorylation detaches tau from microtubules and can lead to the formation of p-tau droplets in neurons. In diseased neurons, stable droplets and aggregates deplete p-tau from the pool available for microtubule binding. This results in uncontrolled microtubule fragmentation, disruption of proteo-homeostasis, cell stress, and eventually neuronal death

AD is the most common cause of dementia. The pathological features of AD are the deposition of diffuse neuritic plaque marked by extracellular amyloid beta (Aβ) and the aggregation of hyperphosphorylated tau protein (p-tau) in cells to form neurofibrillary tangles (NFTs) composed of paired helical filaments [100]. Tau protein is a highly soluble microtubule-associated protein. Phase separation of tau protein can concentrate tubulin and nucleate microtubule bundles and promote assembly of microtubules in healthy neurons [101]. However, the aggregation and deposition of p-Tau protein in AD have neurotoxic effects [102]. Under physiological conditions, LLPS will not appear when tau protein is not phosphorylated. It was found that both hyperphosphorylated tau protein and non-phosphorylated tau protein in the brain of AD represented LLPS. Regardless of its source, the droplets of Tau protein in patients will also become gel-like in minutes and start to spontaneously form tau aggregates over days [100], which are competent in accelerating the progress of AD [103]. Notably, tau rich in Ser and Thr is easily phosphorylated, and phosphorylated tau (p-tau441) is the major driving force for tau LLPS [100, 104, 105]. Moreover, this reaction is driven by a complex combination of electrostatic and hydrophobic interactions within tau protein per se [106]. The acetylation of tau proteins like K3, K18, K259, K290, K321, K353, and S356 inhibited the phase separation of tau proteins, because acetylation eliminates positive charge on Lys residues and weakens intermolecular electrostatic interactions [107]. However, the acetylation of tau at K174 leads to monomeric tau accumulation in vivo and enhances phase separation [108]. Added studies have also identified additional regulators of tau protein phase separation, including heparin, positively charged polyamine, RNA binding proteins, elevated metal ions (like zinc ions), and increased phosphorylation levels [109, 110]. These factors facilitate the formation and aging of tau droplets, and their dynamic decline progresses and evolves into the hardening and deposition of phase separation aggregates as time and temperature increase. Moreover, these neurofibrillary tangles (NFTs) containing filamentous tau protein deposits propagate and further diffuse between neurons. Furthermore, a mutation in the MAPT gene, which codes for tau protein, will result in tau protein misfolding. Thus, an abnormal ubiquitin-proteasome system, improper autophagy degradation system, and an alteration to the ubiquitin protein K290 will all cause the deposition of phase-separated condensate, accelerating the progression of AD [111] (Fig. 2).

### HD

HD is a progressive and fatal neurodegenerative disease; the clinical features include progressive motor dysfunction, cognitive decline, and psychiatric disturbance[112]. HD is caused by a CAG repeat expansion in the huntingtin (HTT) gene. It is caused by a CAG repeat expansion in exon 1 of the HTT gene that encodes an abnormally long polyglutamine (polyQ) tract. This mutated gene is translated to mutant huntingtin (mHTT), the causative agent of the disease [113]. When mHTT is cleaved, the polyglutamine-containing N-terminal fragment is released, and in individuals with HD, this fragment forms inclusions [114]. According to Peskett et al., protein fragments can lead to invertible liquid-like assemblies and transform into solid-like fibrillar assemblies, when the polyglutamine tract arrives at disease-associated lengths [115]. Notably, an N-terminal amphipathic region, a polyQ region, and a proline-rich region constitute huntingtin. mHTT is unstable, and the main form includes monomers, soluble oligomers, and insoluble polymers both in vivo and in vitro [114]. Likewise, polyQ length and duration equally impact how rapidly mHTT oligomers and polymers are shaped from monomers [116]. Moreover, the severity and timing of the onset of HD relate to polyQ region expansion and this change determines whether LLPS occurs in mHTT [117]. Posey et al. reported that purified N-terminal fragments of Huntington protein (Htt-NTF) can be divided into three phases, according to their saturation concentration, size, and shape. M phase is composed of soluble monomers and oligomers. S phase is mainly made up larger soluble aggregates of about 25 nm in diameter and the key components of F phase are insoluble protofibrillar aggregates [118]. Liquid-like condensates of mHTT may have progressed into fibril when polyQ length extends beyond the threshold for HD in vitro [119]. Notably, HTT could interact with several protein arginine methyltransferases (PRMT) through its N-terminal domain [120]. PRMT4 and PRMT6 are the main enzymes specifically methylate HTT and their alteration can cause HTT more difficult to solubilize in cells. Consequently, the extended HTT1-586 fragment can form liquid-like assemblies, which converts to solid assemblies when the R200/205 methylation site is altered, affecting the disease course [119, 120].

### ALS/FTD

ALS is a progressive and fatal neurodegenerative disease characterized by upper motor neurons (UMN), including cranial motor nuclei in the pons and medulla and frontal cortex, and lower motor neurons (LMN), such as motor neurons in the anterior horn of the spinal cord damage, resulting in progressive muscle weakness and atrophy, with fasciculations, hyperreflexia, and spasticity [121, 122]. The pathology is characterized by degeneration and death of motor neurons like pyramidal cells and Baez cells with intracellular inclusion bodies and proliferation of glial cells [122, 123]. Previous studies also showed that mutations in C9orf72, SOD1, TARDBP/TARDBP/TAR DNA binding protein (TDP-43) and fused in sarcoma are the most frequent genetic forms of ALS [124–128]. Other pathogenic mutations include HNRNPA1, HNRNPA2B1, VCP, OPTN, PFN1, ANG, SETX, and MATR3 [127, 129–135]. Moreover, some genetic variants do not cause ALS per se, but enhance ALS susceptibility, such as ATXN2 gene amplification, SMN1 gene duplication, TIA1 mutation, and UBQLN2 mutation [136–138].

Clinically, FTLD is characterized by progressive deficits in behavior, personality, and/or language. The main dramatic behavioral symptoms for this disease include disinhibition, loss of empathy and comprehension, and socially unacceptable behavior [139]. Consequently, relatively localized degeneration of the frontal and temporal lobes is represented in the patient’s brain. Common genetic variants in FTLD patients include MAPT mutation encoding tau protein and C9ORF72 hexanucleotide repeat expansion [140, 141]. Rare variants associated with FTLD include VCP, CHMP2B, TBK1, HNRNPA1, and ATXN2 [142, 143].

ALS and FTLD overlap clinical and neuropathological characteristics, and both are relevant to C9orf72 amplification and RBP gene variants (TDP-43, FUS, ATXN2, EWSR1, TAF15, HNRNPA1, HNRNPA2B1, TIA1, and MATR3) [135, 144, 145]. The underlying mechanisms of these genetic variants are altering the driving force and subcellular localization of protein aggregation which in turn incite abnormal posttranslational modifications (PTMs) and disrupt the protein quality control system, inducing protein misfolding and aggregation.

Diffuse expression and partial pathological clustering of TAR DNA-binding protein 43(TDP-43) and its 35-kd and 25-kd cleavage fragments and FUS proteins are common in the brain of ALS/FTLD patients [146]. The commonly found proteins hnRNPA0, hnRNPA1, hnRNPA2B1, TIA1, ATXN2, PABPC1, and eIF2α in SGs are also present in this pathological RNA binding protein aggregate [12, 147]. Thus, this indicates that SGs are precursors of pathological aggregates. Notably, SGs are complexes composed of ribonucleoprotein particles. Under stressful pressure during physiological states, nuclear RBPs (TIA1, FUS, TDP43, hnRNPA1, hnRNPA2B1, EWSR1, and ATXN2), responsible for regulating mRNA splicing, RNA helicase activity, and RNA polymerase elongation, are transferred to the cytoplasm [148, 149]. After undergoing phase separation to form SG droplets under multivalent interactions between tyrosine residues of their prion-like domains and arginine residues of their RNA-binding domains, it can recruit mRNA and maintain a reversible steady state. SGs can also sort mRNAs and execute mRNA restart, storage, or degradation programs, respectively. Moreover, eIF4G in SGs interacts with TRAF2, thereby avoiding apoptosis and acting as a neuronal protector [150]. When the stress factor is gone, SGs are solubilized with the action of decomposing enzyme such as VCP [151]. Consequently, the internal dynamics of SGs are altered under chronic long-term stress conditions. For example, hyperphosphorylation of mutations in the prion-like domain of TDP-43 prevents nuclear RBPs from returning to the cytoplasm and re-solubilizing SGs. Simultaneously, this process also gives rise to recruit more mRNAs and nuclear RBPs to interfere with the synthesis and transport of RNA by SGs [152, 153]. ALS-linked mutations in the low-complexity structural domains of FUS, hnRNPA2B1, EWS, TAF15, MATR3, and TIA1 lead to increased local concentrations of these proteins, enhancing amyloid interactions and forming pathological aggregations of non-functional oligomers and protofibrils, causing difficulty in degrading both autophagy and ubiquitination pathways, which in turn damages neurons [137, 149, 154]. Furthermore, lipoamide promotes phase separation to generate liquid-like cohesions, thus slowing down the fibrosis progression of FUS proteins and providing a new idea for the treatment of ALS/FTLD from the drive of protein aggregation [155].

**Fig. 3:**
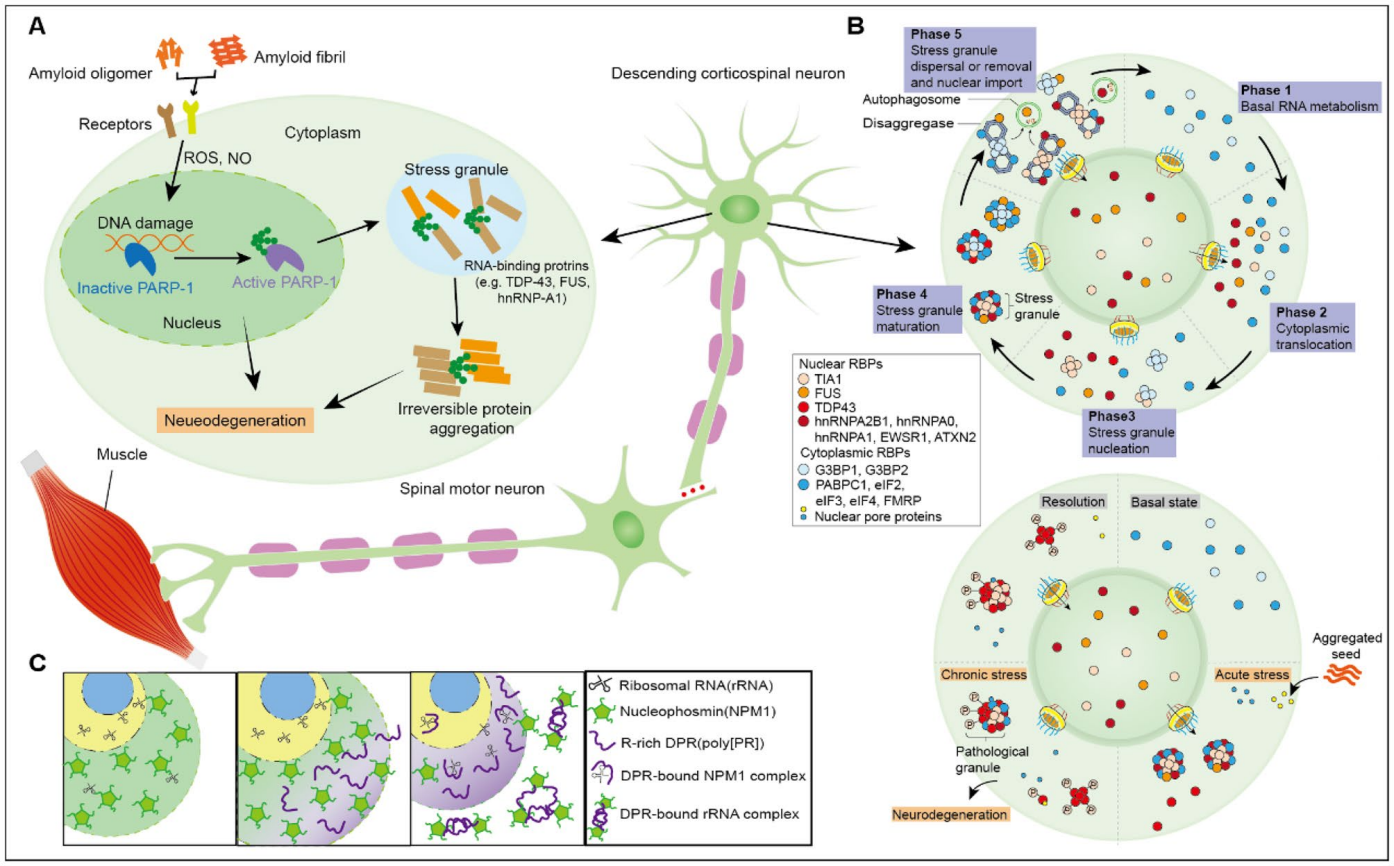
The LLPS in descending corticospinal neurons. **A** In neurodegenerative diseases, accumulation of oxidative stress may induce PARP-1 hyperactivation. In addition, aggregates formed by different amyloid proteins may increase the activity of PARP-1 and the levels of cellular PAR, which together lead to neuronal cell death. **B** Phases in the stress granule cycle. **C** ALS-associated dipeptide repeats (R-rich DPRs) alter NPM1 phase separation, leading to NPM1 sequestration and driving droplet dissolution in vitro and NPM1 delocalization from nucleoli

Altered subcellular localization of RNA-binding proteins can also contribute to disease. Mutations in the nuclear localization signal of FUS lead to protein mislocalization in the cytoplasm [156]. As such, the concentration of FUS in the cytoplasm exceeds the critical value for phase separation, and the concentration of RNA in the cytoplasm is so low that it cannot suffer phase separation properly. Thus, FUS gradually tends to fibrillate [157]. In addition, the cytoplasmic PQC system, unlike the nucleus, lacks nuclear input receptors in the cytoplasm, which cannot specifically bind to the nuclear localization signal in FUS and inhibit its phase separation and aggregation. This results in the accumulation of FUS inclusion bodies in neurons. Repeated amplification of the C9ORF72 gene GGGGCC leads to over-translation of the dipeptide repeat protein poly(proline-arginine) (poly-PR) and the dipeptide repeat protein poly(glycine-arginine) (poly-GR). Poly-PR and poly-GR are extremely toxic and interfere with important components of the nucleolar fluid [158]. The phase separation of nucleophosmin 1 (NPM1) and its abnormal accumulation in the nucleolus accelerate the disease progression of ALS/FTD [159–161]. Poly-PR and Poly-GR, which are rich in arginine, are capable of stronger multivalent interactions with NPM1 and rRNA and also will isolate rRNA and competitively bind NPM1, causing NPM1 to delocalize from the nucleolus [162]. Consequently, disturbances in nucleolus dynamics and organization can inhibit ribosome biogenesis and ultimately lead to cell death. Reducing transcription factor p53 in neurons can reduce poly-PR and poly-GR production and improve axonal degeneration, also providing new ideas for treating ALS/FTD caused by C9ORF72 amplification from inhibiting highly specific transcriptional programs [163]. In addition to pathogenic mutations, abnormal PTMs on RNA-binding proteins also contribute to ALS/FTD-associated pathological phase changes. Aberrant phosphorylation of TDP-43 inclusion bodies is common in sporadic ALS/FTD. Moreover, phosphorylation at TDP-43 serine residues 409 and 410 promotes TDP-43 phase separation, but FUS phosphorylation inhibits its phase separation [164]. The binding of TDP-43 and FUS to poly (ADP-ribose) (PAR) on RNA-binding proteins promotes the phase separation of FUS, TDP-43, and hnRNP A1 [165]. More importantly, PAR binding promotes the recruitment and targeting of TDP-43 and hnRNPA1 to SGs, causing excessive accumulation of RBPs in SGs and accelerating SG sclerosis [166]. Furthermore, UBQLN2 is extensively involved in PQC as a ubiquitin-bridging egg [167]. Mutant UBQLN2 prevents the misfolded protein from binding to Hsp70, resulting in impaired targeting of the proteasome, thereby preventing degradation of the misfolded protein via the ubiquitination pathway. Thus, the misfolded protein concentration exceeds the phase separation threshold to form cytotoxic amyloid fibrils [111] (Fig. 3).

### PD

**Fig. 4:**
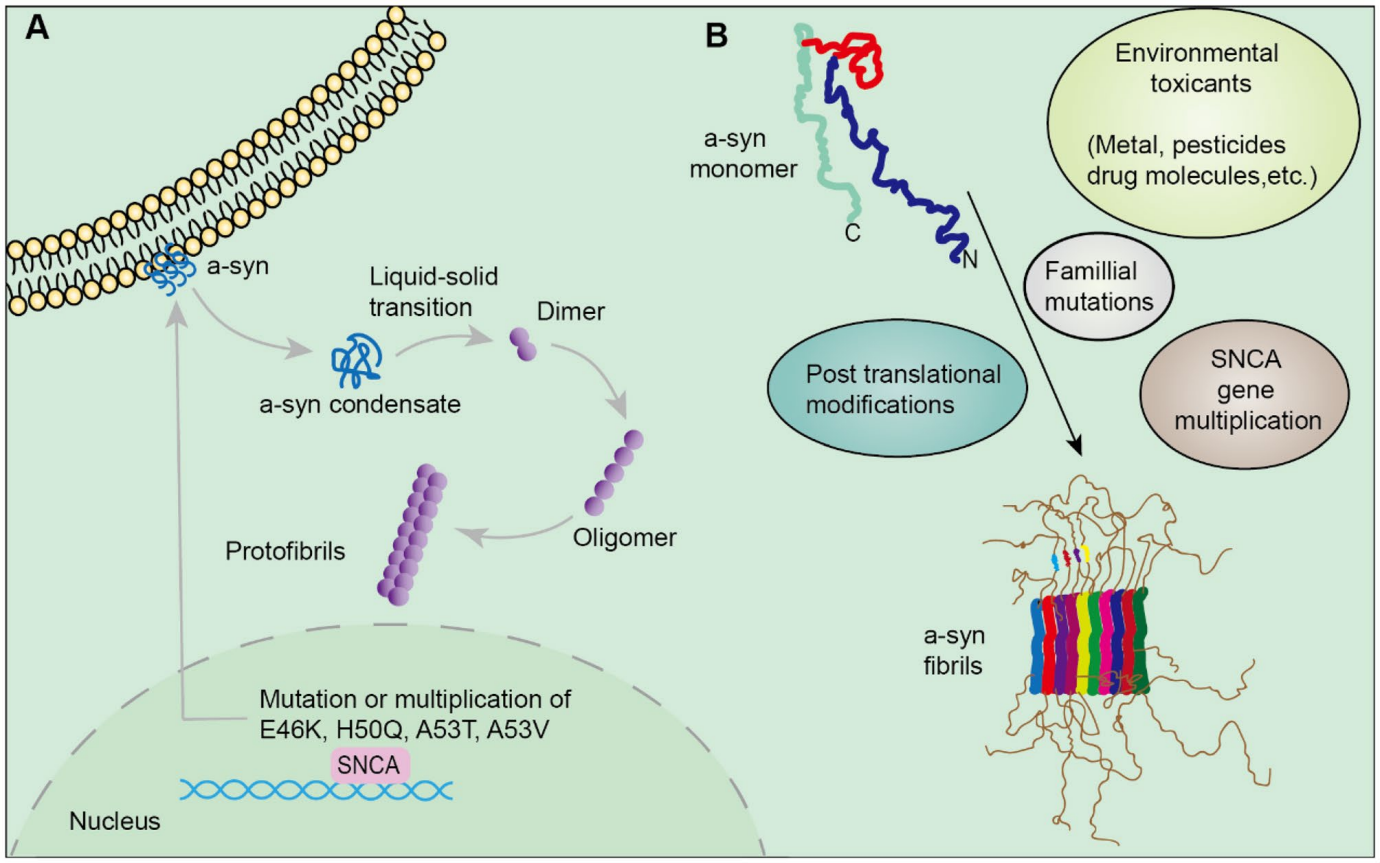
The process of α-syn aggregation. **A** When mutations occur in A53T, E46K, H50Q, and A53V, the balance between α-syn generation and clearance is disrupted and the monomers aggregate to form oligomers. The oligomers also tend to form protofibrils and eventually fibrils. **B** Factors modulating misfolding and aggregation of a-Syn

Parkinson’s disease (PD) is a long-term, progressive disease of the central nervous system that is characterized by a movement dysfunction brought on by a particular type of dopamine (DA) neuron degeneration in the substantia nigra pars compacta [168]. The substantia nigra and striatum lost dopaminergic neurons, which decreased from 550,000 to 100,000, and the current findings may also be an indication of decreased GABA production. These are the key neuropathological characteristics of PD, and all of them reduce the formation of non-motor and motor symptoms and have a negative impact on patient quality of life [169]. The Lewy body (LB), a cytoplasmic aggregate of α-synuclein (αSyn), was considered the gold standard for definitive diagnosis [97]. The phase separation and droplet formation of αSyn in physiological conditions have been demonstrated, and this may be one of the key factors underlying Sal’s neuroprotective properties [170, 171]. A53T, E46K, H50Q, and A53V are four missense point mutations in the SNCA gene, producing αSyn, which have been correlated to family types of PD [172, 173]. It can also be detected that αSyn condensate undergoes a liquid-to-solid phase transition. As such, they form cytotoxic αSyn oligomers and protofibrils, leading to neuronal dysfunction and cell death [171]. αSyn has three major domains: the N-terminal, which can bind to lipids such as membranes and PEGs; the C-terminal, which is rich in negative charges; and the hydrophobic non-amyloidal component region (NAC), which is the core region for the formation of αSyn oligomers and protofibrils [174]. Under physiological conditions, the N-terminal and NAC low-complexity domains interact multivalently [175], driving αSyn to experience spontaneous phase separate, accumulating in the presynaptic membrane, and playing an important role in vesicle transport [176]. Under pathological conditions (high critical concentration and/or presence of factors that promote spontaneous α-Syn phase separation), αSyn condensate frequently undergoes liquid-solid phase transition [174]. Acidic environments, higher concentrations of metal cations [18, 177, 178] (e.g., copper, manganese, calcium, and trivalent iron) and liposomes [179], and abnormal PTMs, mainly Ser129 phosphorylation (locate at the C-terminus) [180], promote the progressive formation of αSyn dimers, oligomers, and protofibrils. Moreover, the interaction of αSyn with tau has been shown to synergistically promote fibrosis [180]. The C-terminus of αSyn has been detected in some PD patients to bind to the proline-rich P2 region of tau, while mutations in the SNCA gene (mainly at the N-terminal) may promote phase separation by enhancing the sensitivity of αSyn to the harmful environment [181]. Surprisingly, αSyn oligomers are the key substance causing neuronal damage, and misfolded αSyn oligomers that are phagocytosed by normal neurons can act as a template to induce misfolding [171]. Additionally, αSyn can reduce ferrous ions to ferric ions and redistribute iron ions, leading to iron metabolism disorders, causing cellular oxidative stress and neuronal death [182]. Unfortunately, there is no curative approach to PD. However, researchers have now constructed and optimized the structure of a small ubiquitin-like modifier (SUMO)1 variant to obtain SUMO1, the segment including the hydrophobic binding pocket residues 15–55 [183], to interact multivalently with two SUMO-interacting motifs (SIM) located in the control of αSyn to exert stronger phase separation, inhibit αSyn aggregation, and improve the pathological manifestation of PD (Fig. 4).

## Conclusion

This review focuses on several neurodegenerative diseases in which we comprehensively expound the underlying mechanism of phase separation involved in normal physiological activities and the occurrence of human disease [184]. Overall, aberrant phase separation can lead to the formation of aggregates, likely accelerating disease progression. Yet, there are still issues that have not been resolved. For example, how we could prevent phase separation from happening with disease progression remains unanswered. Phase separation mainly takes place within cells. However, the identification of phase separation becomes a major challenge due to the complexity of intracellular components. In addition, the indigenous milieu for these cells in vivo was not entirely replicated by the in vitro environment. Thus, data obtained in vitro should be validated with in vivo experiments, which will contribute to our understanding of the role of phase separation in neurodegenerative diseases. Notably, phase separation detection technology essentially restricts us from understanding mechanisms in vitro and in vivo. There are still several horizons for the development of phase separation in the field of physiological and pathological processes, as well as the development of targeted drugs. Therefore, we believe that with the continuous exploration and progress in many biomedical disciplines, the mystery of phase separation will eventually be fully unraveled and applied.

## Data Availability

Not applicable.
